# On the Structure and Development of the Liver

**Published:** 1848-06

**Authors:** C. Handfield Jones

**Affiliations:** M.B. Cantab.


*On the Structure and Development of the Liver.* By C. HANDFIELD JONES, M.B. Cantab. Communicated by Sir BENJAMIN C. BRODIE, Bart., F.R.S. &c.—The author gives a detailed description of the structure of the liver in animals belonging to various classes of the animal kingdom. He states that in the Bryozoon, a highly organized polype, it is clearly of the follicular type; and that in the Asterias, the function of the liver is probably shared between the closed appendage of the stomach, and the terminal cæca of the large ramifying prolongations of the digestive sac contained in the several rays. Among the Annulosa, the earthworm presents an arrangement of the elements of the hepatic organ, corresponing in simplicity with the general configuration of the body, a single layer of large biliary cells being applied as a kind of coating over the greater part of the intestinal canal. In another member of the same class, the Leech, in which the digestive cavity is much less simple, and presents a number of sacculi on each side, these elements have a very different disposition; and the secreting cells, although some remain isolated, for the most part coalesce to form tubes, having a succession of dilatations and constrictions, and finally uniting and opening into the intestine. In Insects, the usual arrangement is that of long curved filamentary tubes, which wind about the intestine; these, in the meat-fly, are sacculated throughout the greater part of their course, till they arrive quite close to the pylorus, where they open; near their origin they appear to consist of separate vesicles, which become gradually fused together, but occasionally they are seen quite separate. The basement membrane of the tubes is strongly marked, and incloses a large quantity of granular matter of a yellowish tinge, with secreting cells; another portion of the liver consists of separate cells lying in a granular blastema, which cells, in a later stage of development, are seen to be included in vesicles or short tubes of homogeneous membrane, often coalescing and exhibiting a more or less manifestly plexiform arrangement. The author has termed this the *Parenchymatous portion* of the liver, on account of its general appearance and mode of devolopment, though he has not been able to determine whether the tubes always originate from it. Among the Arachnida, the follicular type of arrangement prevails; and the same is the case with the Crustacea, the follicles in these last being distinctly visible to the naked eye. In Mollusca also, we find the follicular arrangement universally to obtain; yet in certain cases the limiting membrane of the follicles cannot be shown to exist, and the author therefore thinks that its importance is probably not great, but that it serves chiefly to fulfil the animal function which its synonym, "*basement*" indicates. The quantity of retained secretion in the liver of molluscs seems clearly to imply that the bile in them is not an excrementitious fluid; it is used slowly on account of the imperfect character of the respiration.

In passing from the Invertebrata to the Vertebrate division of the animal kingdom, and beginning with the class of Fishes, a great change is immediately manifest in the form and character of the biliary organ; it is now a gland of solid texture, to which the term *parenchymal* is justly applied. Two portions may be distinguished in it, namely, the secreting parenchyma, consisting of delicate cells, or very often of nuclei, granular and elaborated matters in great part, and the excreting ducts, which though completely obscured by the surrounding bulky parenchyma, may yet be satisfactorily demonstrated, and traced often to their terminal extremities in the following manner. If a branch of the hepatic duct be taken up in the forceps, it may be dissected out without much difficulty from the surrounding substance, which is very soft, and yields readily to gentle manipulation; when a trunk is in this way removed and placed under the microscope, a multitude of minute ramifications are seen adhering to it; among these not a few may be discovered, which do not appear to have suffered injury; some are occassionally seen terminating by distinctly closed extremities; more usually the duct becomes very minute, and gradually loses all definite structure, appearing at last like a mere tract of granular matter; in either case there is no communication by continuity with the surrounding parenchyma. Large yellow corpuscles, peculiar cells, and a considerable quantity of free oily matter usually existing in the liver of various fishes, seem generally to indicate a great superiority in the amount of secretory over that of excretory action, and to betoken clearly the feeble intensity of the aerating functions.

In reptiles, there is the same arrangement of the liver, namely, a secreting parencyma of cells and an apparatus of excretory ducts, which have the same essential characters as those of fishes; but there exists very frequently in the parenchyma remarkable dark corpuscles, which appear to be masses of retained biliary matter, the import of which, in the situation they occupy, is doubtless the same as that of the similar masses existing in fishes.

In Birds, the parenchyma of the liver is remarkably free from oily or retained biliary matters; it often consists almost wholly of free nuclei and granular matter, with scarcely a single perfect cell; the excretory ducts often greatly resemble those of reptiles, sometimes rather those of mammalia; the essential character is, however, always the same, namely, that they terminate without forming any important connection with the parenchyma.

In mammalia, the parenchyma of the liver consists usually of perfect cells, which are arranged often in linear series of considerable length, radiating from the axis of each lobule; these unite at various points with each other, so as to present a more or less decidedly plexiform appearance. Each lobule, as described by Mr. Kiernan, is separated from the adjacent ones by the terminal twigs of the portal vein, and to a greater or less extent by a "fissure," though in most animals the lobules are continuous with each other both above and below the fissure. The elaboration of the secreted product seems to be most completely effected in the cells adjoining the margins of the lobules, which are often seen to contain a larger quantity of biliary matter than those in the interior, and to be apparently in the act of discharging it into the fissure; the margin of the lobule then presents an irregular surface with large globules of the secretion clustering together all over it. The capsule of Glisson surrounding the vessels in the portal canals, gives a fibrous investment to those surfaces of the lobules which are toward the canal; but when it has arrived in the fissures, it forms a continuous membrane lining the surfaces of opposite lobules; this membrane is often truly homogeneous and closely resembles the basement tissue; there appears occasionally to be a delicate epithelium on its free surface; but this, as well as the membrane itself, is often absent when the margin of the lobules is in that condition which has just been described and which may be termed *active.* The minute branches of the hepatic duct, as they approach their termination, undergo a remarkable alteration in their structure; they lose their fibrous coat, which blends itself with the membranous expansions of the capsule of Glisson; their basement membrane becomes gradually indistinct, and at last ceases to exist; and the epithelial particles no longer retain their individuality, but appear to be reduced to mere nuclei, set very clos etogether in a faintly granular basis substance. The mode of their termination is not uniformly the same; frequently they present distinctly closed rounded extremities, between one and two thousandths of an inch in diameter; at other times they seem to cease gradually in the midst of fibrous tissue, the nuclei alone being disposed for some little way in such a manner as to convey the idea of a continuation of the duct. These ducts can seldom be discerned in the fissures, but have several times been seen in the "spaces," where several fissures unite; they do not form anything like a plexus between the lobules. From the anatomical relation of the ducts to the parenchyma, and from the circumstance that a distinct vessel conveying a different kind of blood is distributed to the hepatic duct, as soon as the liver assumes the parenchymal form, it seems probable that the mode in which the secreted bile is conveyed out of the organ, is by permeating the coats of the minute ducts in obedience to an endosmotic attraction, which takes place between the bile in which the ducts may be said to be bathed, and a denser (perhaps mucous) fluid formed in their interior. The large quantity of oily matter frequently existing in a free statein the secreting parenchyma of the liver, which must be regarded as a product of secretory action, seems to suggest the idea, that a certain quantity of the biliary secretion may be directly absorbed into the blood, and in this manner conveyed away from the organs, just as occurs in the thyroid body, suprarenal capsules, and other glands unprovided with efferent ducts.

With respect to the development of the liver, the author considers the opinion of Reichert to be decidedly the correct one, namely, that its formation commences by a cellular growth from the germinal membrane, independently of any protrusion of the intestinal canal. On the morning of the fifth day, the œsophagus and stomach are clearly discernible, the liver lying between the heart, which is in front, and the stomach, which is behind; it is manifestly a parenchymal mass, and its border is quite distinct and separate ftom the digestive canal; at this period the vitelline duct is wide, it does not open into the abdominal cavity, but its canal is continued into an anterior and posterior division, which are tubes of homogeneous membrane, filled, like the duct, with opaque oily contents; the anterior one runs forwards, and forms behind the liver a terminal expanded cavity, from which there passes one offset, which gradually dilating, opens into the stomach; a second, which runs in a direction upwards and backwards, and forms apparently a cæcal prolongation; and the third and fourth, which are of smaller size, arise from the anterior part of the cavity and run to the liver, though they cannot be seen to ramify in its substance; at a somewhat later period, these offsets waste away, excepting the one which is continued into the stomach, and then the mass of the liver is completely free and unconnected with any part of the intestine. As the vitelline duct contracts, the anterior and posterior prolongations of it become fairly continuous and form a loop of intestine, the posterior division being evidently destined to form the cloaca and lower part of the canal. The final development of the hepatic duct takes place about the ninth day, by a growth proceeding from the liver itself, and consisting of exactly similar material; this growth extends towards the lower part of the loop of the duodenum, which is now distinct, and appears to blend with the coats of the intestine; around it, at its lower part, the structure of the pancreas is seen to be in process of formation. The further progress of development of the hepatic duct will, the author thinks, require to be carefully examined, but the details he has given in this paper have satisfied him of the correctness of the statement that the structure of the liver is essentially parenchymal.—*Proceedings of the Royal Society*, 1847.

